# DC-SIGN Polymorphisms Associate with Risk of Hepatitis C Virus Infection Among Men who Have Sex with Men but not Among Injecting Drug Users

**DOI:** 10.1093/infdis/jix587

**Published:** 2017-11-13

**Authors:** Gaby S Steba, Sylvie M Koekkoek, Joost W Vanhommerig, Kees Brinkman, David Kwa, Jan T M Van Der Meer, Maria Prins, Ben Berkhout, Michael Tanck, William A Paxton, Richard Molenkamp, Janke Schinkel, MSM Observational Study of Acute Infection with Hepatitis C (MOSAIC) Study Group and Amsterdam Cohort Studies (ACS)

**Affiliations:** 1 Department of Medical Microbiology, Academic Medical Center, Amsterdam, The Netherlands; 2 Department of Infectious Diseases, Public Health Service of Amsterdam, Amsterdam, The Netherlands; 3 Department of Internal Medicine, Amsterdam, The Netherlands; 4 Department of Microbiology, Onze Lieve Vrouwe Gasthuis, Amsterdam, The Netherlands; 5 Department of Internal Medicine, Division of Infectious Diseases, Tropical Medicine, and AIDS, Center for Infection and Immunity Amsterdam, Amsterdam, The Netherlands; 6 Department of Clinical Epidemiology, Biostatistics and Bioinformatics, Academic Medical Center, Amsterdam, The Netherlands; 7 Department of Clinical Infection, Microbiology and Immunology, Institute of Infection and Global Health, University of Liverpool, UK

**Keywords:** Hepatitis C virus, lectin, DC-SIGN, single nucleotide polymorphism, sexual transmission

## Abstract

We aimed to identify whether genetic polymorphisms within L-SIGN or DC-SIGN correlate with hepatitis C virus (HCV) susceptibility. A men who have sex with men (MSM) and an injecting drug users (IDU) cohort of HCV cases and multiple-exposed uninfected controls were genotyped for numerous L-SIGN and DC-SIGN polymorphisms. DC-SIGN single nucleotide polymorphisms (SNPs) −139, −871, and −939 correlated with HCV acquisition in the MSM cohort only. When the same SNPs were introduced into a transcription activity assay they demonstrated a reduction in expression with predicted alteration in binding of transcription factors. DC-SIGN promoter SNPs correlated with risk of HCV acquisition via sexual but not IDU exposure, likely through modulation of mRNA expression levels.

## Teaser

We identified a number of single nucleotide polymorphism (SNPs) in the DC-SIGN promoter region associate with risk of hepatitis C virus transmission via sexual exposure but not via intravenous drug use, and that the SNPs decreased promoter activity.

Hepatitis C virus (HCV) represents a major global health burden, with 350000 people dying annually from HCV-related liver disease [1]. Intravenous drug use is now the major transmission route. Nevertheless, since 2000, sexual transmission has been reported frequently among HIV-infected men who have sex with men (MSM) and is associated with high-risk sexual behavior. Interestingly, some individuals remain uninfected despite practicing high-risk behavior(s). Studies have shown that ultimately 10%–20% of injecting drug users (IDU) do not seroconvert, suggesting a biological reason why some individuals are less prone to contract HCV [2].

DC-SIGN (dendritic cell specific ICAM-grabbing nonintegrin, CD209) and L-SIGN (DC-SIGN related, CD209L) are c-type lectins, which have been suggested to play a role in HCV transmission and infection [3]. DC-SIGN is a calcium-dependent cell-surface lectin of dendritic cells (DCs) [4]. DCs are localized in skin and mucosal tissues and may serve as a replication reservoir for HCV [4, 5]. L-SIGN is mainly expressed on liver and lymph node sinusoidal endothelial cells. It shares 77% amino acid identity with DC-SIGN and it has been shown to capture several viruses, including HCV [3]. Whereas the DC-SIGN neck region on exon 4 is highly conserved (7 repeats in the majority of individuals) the L-SIGN neck region is very variable [6]. This repeat region has been suggested to affect disease susceptibility and outcome for HIV-1 infection [7–9].

The objective of this study was to analyze the frequency of previously reported genetic variations in DC/L-SIGN genes in individuals from 2 well-defined cohorts at risk of HCV infection who either seroconverted or remained uninfected. We identified 3 DC-SIGN SNPs that were associated with HCV susceptibility through high-risk sexual exposure but not with IDU. Furthermore, we assessed whether these SNPs in the DC-SIGN promoter affect its activity.

## METHODS

### Study Populations

#### MSM Cohort (MOSAIC)

Sixty-two HIV-1 infected MSM participating in the MSM Observational Study of Acute Infection with Hepatitis C (MOSAIC) cohort were included. Risk behavior data was available from behavioral questionnaires collected at 6-month intervals and MOSAIC Risk Scores were calculated [10]. Participants were categorized as multiple exposed uninfected (MEU, n = 30) or multiple exposed infected (MEI, n = 32) based on reported behavioral risk factors at inclusion or any of the follow-up visits, which have been shown to be associated with increased risk of acquiring HCV sexually [10, 11]. The MOSAIC study was approved by the Institutional Review Board of the Academic Medical Center under assigned study numbers NL26485.018.09 and NL48572.018.14.

#### IDU Cohort (Amsterdam Cohort Studies)

Sixty-two participants from the Amsterdam Cohort Studies (ACS) among IDUs were selected, who started injecting drugs intravenously before 1990, a period of high HCV incidence (up to 27.5/100 person years) [12]. The ACS among IDUs was an open prospective cohort study recruiting drug users between 1985 and 2016 investigating the epidemiology, natural history, and pathogenesis of HIV-1 infection and other blood-borne and/or sexually transmitted diseases. Participants who injected more than 2 years and remained HCV seronegative during follow-up (n = 40) were classified as MEU whereas 22 MEI seroconverted for HCV during follow-up. Total duration of injecting drugs and follow-up was similar for MEU and MEI (Supplementary Table S1). During follow-up no statistical difference was found between MEI and MEU when comparing needle-sharing events. The ACS study was approved by the Institutional Review Board of the Academic Medical Center under assigned study numbers MEC 07/182 and MEC 09/040.

### DNA Isolation and Genotyping

DNA was isolated from 200 µL participant serum utilizing the QIAamp DNA blood mini kit (Qiagen). The number of repeat domains within the L-SIGN repeat region was determined for each subject by polymerase chain reaction (PCR). PCR reactions contained 5 µL of template DNA, 400 nM forward primer, 400 nM reverse primer, 2.5 mM MgCl_2_, 0.2 mM dNTPs, 0.1 mg/mL bovine serum albumin (BSA), 1.25 units FastStart Taq DNA polymerase in a total volume of 25 µL 1 × Faststart PCR buffer.

L-SIGN SNP rs2277998 was assessed using the Ready-to-use hot start reaction mix for High Resolution Melting (HRM) curve analysis using the LightCycler® 480 (Roche). The reaction contained 2.0 µL DNA template, 2.5 mM MgCl_2_, 8 ng α-casein, 450 nM forward primer (Biolegio) and 450 nM reverse primer (Biolegio) in a total volume of 20 µL 1 × HRM master mix.

To assess reported DC-SIGN SNPs in the promoter region at positions −939 (rs735240), −871 (rs735239), −336 (rs4804803), and −139 (rs2287886), a DNA fragment covering approximately 1000 bp upstream of the ATG translation start site was amplified with 2 primer sets. The amplicons were sequenced in both directions with the same primers using Big Dye Terminator according to manufacturer’s instructions (Applied Biosystems, Inc., Norwalk, CT). Primers and amplification conditions are summarized in Supplementary Table S2.

### Cell Culture

HEK 293T/17 cells (ATCC number: CRL-11268) were cultured in Dulbecco’s Modified Eagle’s Medium (DMEM; Invitrogen) supplemented with 10% FCS, 1 × MEM nonessential amino acids (Gibco), 100 U/mL penicillin, and 100 U/mL streptomycin. Cells were incubated at 37°C in 5% CO_2_ and passaged twice a week upon 90% confluence.

### Construction of DC-SIGN Promoter Variants

The DC-SIGN promoter variants were constructed by amplifying the DC-SIGN promoter region using DNA from 1 study participant with the −139A, −871A, and −939G variants using primers tailed with *XhoI* and *HindIII* restriction sites. The amplicons were cloned into the pGL10.4 vector[luc2] (Promega) at the *XhoI* and *HindIII* sites. Promoter variants (see Supplementary Figure S1) were established by site directed mutagenesis. Mutations were made with the QuikChange II Site-Directed Mutagenesis Kit (Agilent Technologies) with specific mutagenic primers (Supplementary Table S2).

### Transfection of 293T Cells With Promoter Constructs and Analysis of Luciferase Expression

293T/17 cells were transfected with the DC-SIGN promoter constructs and a *Renilla* luciferase expression plasmid (pRL-CMV) (Promega) for normalization in a 50:1 ratio using Xtremegene (Invitrogen). Cells were incubated 24 hours and lysed with Passive Lysis Buffer (Promega). Lysate (5 µL) was used to measure firefly and *Renilla* luciferase activity with Dual-Glo luciferase assay system (Promega).

### Prediction of Transcription Factor Binding Sites

Transcription factor (TF) binding sites were predicted using the PROMO database (http://alggen.lsi.upc.es/) which uses TRANSFAC for prediction [13].

### Statistical Analysis

DC/L-SIGN SNP genotype frequencies between MEU and MEI were compared using logistic regression. Initially, an additive/dominance deviation joint 2 degrees of freedom test (with 2 genotype-dependent variables in the regression, one with 0/1/2 coding and the second with 0/1/0 coding) was carried out. Subsequently, in case of dominance deviation (*P* < .1), a dominant or recessive genetic model was assumed, otherwise an additive genetic model was assumed in the logistic regression model used to estimate the odds ratio (OR) and corresponding 95% confidence interval (CI). A *P* value < .05 was considered statistically significant and all analyses were carried out using SPSS software (IBM, version 20).

## RESULTS

### DC-SIGN −139GG, −871GG, and −939AA Are Associated With Reduced HCV Susceptibility in MSM

Patient characteristics are summarized in Supplementary Table S1. In the MSM cohort, 3 DC-SIGN SNPs were significantly associated with HCV infection (Table 1). The −139GG was found more frequently in MEU (63.3% in MEU compared to 37.5% in MEI). Additionally, −871GG (36.7% in MEU compared to 12.5% in MEI) and the −939AA (53.3% in MEU compared to 21.9% in MEI) were found more often in MEU, indicating that −139GG, −871GG, and −939AA genotypes protect against HCV acquisition (OR, 0.35; *P* = .045; OR, 0.23 *P* = .027; and OR, 0.23 *P* = .009, respectively). The −336 SNP was not significantly associated with HCV susceptibility.

**Table 1.** Distribution of DC/L-SIGN Single Nucleotide Polymorphism in Multiple Exposed Infected (MEI) and Multiple Exposed Uninfected (MEU) Individuals

	MEI vs MEU							95% CI	*P* value
	genotype	MEI (n)	MEI (%)	MEU (n)	MEU (%)	Dominance deviation^a^	OR		
**MOSAIC**									
**L-SIGN rs2277998**	AA	1	3%	4	13%			0.29 to 2.37	.73
	AG	11	34%	6	20%	0.09^b^	0.83		
	GG	20	63%	20	67%		(GG vs AG+AA)		
**DC-SIGN −139**	AA	3	9%	6	20%			0.12 to 0.97	.04^c^
	AG	17	53%	5	17%	0.01^b^	0.35		
	GG	12	38%	19	63%		(GG vs AG+AA)		
**DC-SIGN −336**	AA	22	69%	25	83%			0.74 to 7.32	
	AG	9	28%	5	17%	0.99	2.32		
	GG	1	3%	0	0%		(per G allele)		
**DC-SIGN −871**	AA	16	50%	12	40%			0.06 to 0.85	.03^c^
	AG	12	38%	6	20%	0.08^b^	0.23		
	GG	4	13%	11	37%		(GG vs AG+AA)		
**DC-SIGN −939**	AA	7	22%	16	53%			0.07 to 0.69	.01^c^
	AG	18	56%	5	17%	<0.01^b^	0.23		
	GG	7	22%	8	27%		(AA vs AG+GG)		
**ACS**									
**L-SIGN rs2277998**	AA	1	4,50%	2	5%			0.44 to 2.56	.896
	AG	10	45,50%	17	43%	0.85	1.06		
	GG	11	50,00%	21	53%		(per A allele)		
**DC-SIGN −139**	AA	5	22,70%	10	25%			0.36 to 1.38	.3
	AG	5	22,70%	16	40%	0.22	0.70		
	GG	12	54,50%	14	35%		(per A allele)		
**DC-SIGN −336**	AA	15	68,20%	28	70%			0.55 to 2.60	.65
	AG	4	18,20%	9	23%	0.50	1.20		
	GG	3	13,60%	3	8%		(per G allele)		
**DC-SIGN −871**	AA	13	59,10%	18	45%			0.36 to 1.74	.56
	AG	6	27,30%	18	45%	0.21	0.79		
	GG	3	13,60%	4	10%		(per G allele)		
**DC-SIGN −939**	AA	4	18,20%	7	18%			0.42 to 1.79	.71
	AG	8	36,40%	18	45%	0.56	0.87		
	GG	10	45,50%	15	38%		(per A allele)		

The rs2287886 GG, rs735240 AA and rs735239 GG genotypes are significantly associated with protection against hepatitis C virus acquisition in the MOSAIC (men who have sex with men) cohort but no significant associations within the ACS (injecting drug users) cohort.

^a^ *P* value of dominance deviation test.

^b^Dominance deviation *P* value < .1.

^c^Statistically significant (<.05).

As a statistically significant difference was found in the baseline Mosaic Risk Score between MEU and MEI, a sensitivity analysis was done, including only participants with a MOSAIC Risk Score ≥ 2. The association became stronger for all 3 SNPs (−139, −871, and −939), with strong statistical significance for SNP −871 and SNP −939 (Supplementary Table S3). In the ACS IDU cohort, no significant associations were found between SNPs and HCV susceptibility.

### No Associations Between L-SIGN Polymorphisms and HCV Susceptibility

No association with HCV susceptibility was found for L-SIGN SNP rs2277998. In addition, the L-SIGN repeat distribution between MEI and MEU was similar for both cohorts (Supplementary Table S4). No significant difference in zygosity for the L-SIGN repeat was found between MEI and MEU (OR, 0.982 *P* = .961) (Supplementary Table S5).

### DC-SIGN SNPs Affect Promoter Activity

**Figure 1.**
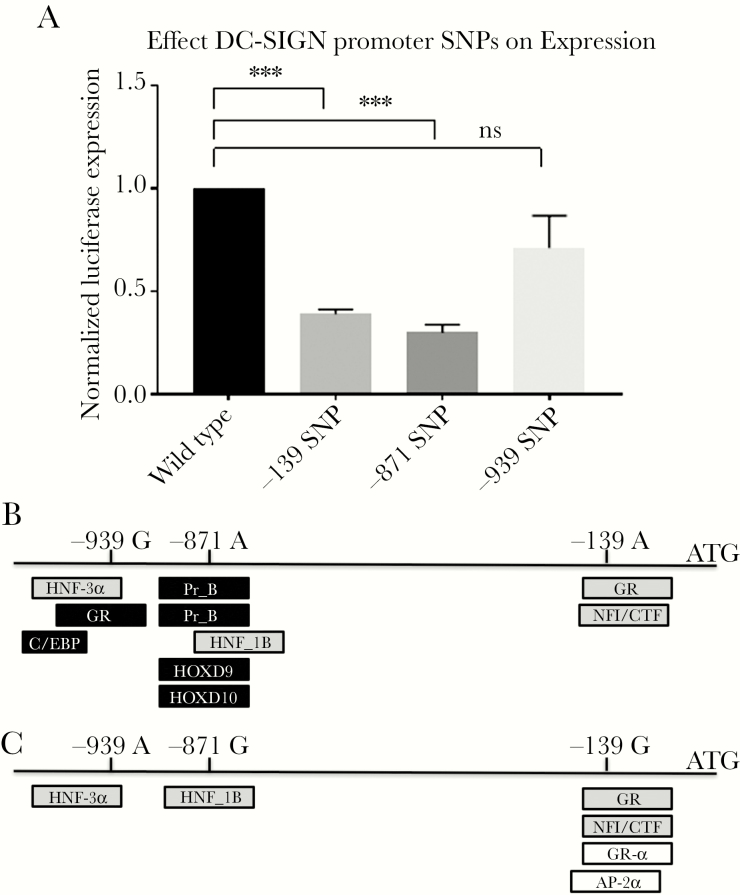
Effect of single nucleotide polymorphisms (SNPs) on DC-SIGN promoter activity. A, The −139 SNP causes a reduction of 2.6 fold (P = .0005), the −871 SNP of 3.3 fold (P = .0009), and the −939 SNP a 1.4 fold (ns, not significant) (***P < 0.001). B, Protective SNPs affect transcription factor (TF) binding sites in the DC-SIGN promoter. Putative binding of TFs to DC-SIGN promoter sequences without (B  ) and with (C  ) protective SNPs. Some TFs do not bind anymore to the sequence containing protective SNPs (black), some bind both sequences (grey), and some bind exclusively to the SNP containing the protective variant (white).

We tested the effect of the promoter variants within the DC-SIGN promoter on transcription activity by using luciferase promoter constructs (Supplementary Figure S1). The −139G caused a 2.6-fold reduction (*P* < .001), the −871G a 3.3-fold reduction (*P* < .001), and the −939A a 1.4-fold reduction (*P* = .086) (Figure 1A). These data suggest that the DC-SIGN promoter variants affect transcription levels and thereby protein and cell surface expression patterns. We investigated whether the observed decrease in DC-SIGN promoter activity for specific SNPs could be due to alterations in TF binding sites, by a in silico comparison of predicted TF binding sites of promoter variants (Figure 1B). The variants at the −139, −871, and −939 sites do affect multiple predicted TF binding sites, with some putative sites lost (GR, C/EBP, Pr-B, Pr-A, HOXD9, HOXD10) and some TF binding sites gained (GR-Alpha, AP-2Alpha). This would indicate that the SNPs identified within the DC-SIGN promoter region can modulate activity through differential binding of transcription factors.

## DISCUSSION

Here we investigated whether polymorphisms in DC-SIGN and L-SIGN correlated with susceptibility to HCV infection in 2 well-defined cohorts consisting of individuals at high risk of HCV infection through sexual or intravenous exposure. We selected polymorphisms based on what has been reported within the literature for HCV as well as other infectious agents. In the MSM cohort we identified an association of HCV susceptibility with 3 DC-SIGN SNPs. These SNPs were not associated with HCV susceptibility in the IDU cohort. No effects were found for the DC-SIGN −336 SNP, the L-SIGN SNP rs2277998, and repeat polymorphism in either cohort.

We studied 4 SNPs in the DC-SIGN promoter region, of which 3 (−139, −871, and −939) were found to correlate with HCV susceptibility in MSM, with −139G showing the strongest effect. Although the same SNPs have previously been associated with other infectious diseases, this is the first time SNPs have been reported to be associated with susceptibility to sexual transmission of HCV. Interestingly, the −139G SNP has also been reported to protect against sexual transmission of HIV-1 [14].

It has been published previously that the combination of −139G and −939A in the DC-SIGN promoter region significantly reduces DC-SIGN expression on immature DCs compared to −139A and −939G [15]. We now show that the −139G and −871G SNP independently cause a reduction in promoter activity, while the −939A variant failed to reach statistical significance (*P* = .085). The DC-SIGN promoter encodes multiple TF binding sites, which are in silico predicted to be affected by the −139, −871, and −939 variants. This strongly suggests that the decreased promoter activity observed in vitro is (at least partly) caused by a reduction in TF binding, which will require further testing.

As our study was small and HCV exposure may have been lower in the uninfected study groups, our observations clearly need to be confirmed in larger cohorts. However, the functional data supports the associations of the SNPs with protection against HCV acquisition. Collectively, our data suggest that DC-SIGN plays a role in HCV acquisition via sexual and not intravenous exposure. This effect appears to be mediated by reduced DC-SIGN expression, suggesting that DC-SIGN on DCs plays a role in sexual transmission of HCV, similar to its role in HIV infection [4]. We hypothesize that DCs transfer HCV to the liver through DC-SIGN; individuals with the protective genotypes will have lower DC-SIGN expression, resulting in a reduced susceptibility to sexual acquisition of HCV. Alternatively, DC-SIGN expression on DCs at mucosal surfaces may influence HCV antigen capture and induction of localized immune responses and modulate mucosal protection against HCV acquisition, which does not play a role in intravenous exposure. Further studies into the exact mechanism behind DC-SIGN affecting HCV infection susceptibility are warranted to better understand how DC-SIGN expression levels might influence immune responses, as well as mechanisms of transmission.

## Supplementary Data

Supplementary materials are available at *The Journal of Infectious Diseases* online. Consisting of data provided by the authors to benefit the reader, the posted materials are not copyedited and are the sole responsibility of the authors, so questions or comments should be addressed to the corresponding author.

Supplementary Table S1

Supplementary Table S2

Supplementary Table S3

Supplementary Table S4

Supplementary Table S5

Supplementary Figure S1

Supplemental Legends

## Notes

***Acknowledgments.*** The authors would like to thank participants of the ACS and MOSAIC study, Margreet Bakker for sample storage and handling, and Astrid Newsum for data handling. This work was conducted within the framework of the Amsterdam Cohort Studies on HIV infection and AIDS (www.amsterdamcohortstudies.org), a collaboration between the Public Health Service of Amsterdam, the Academic Medical Center of the University of Amsterdam, Sanquin Blood Supply Foundation, the University Medical Center Utrecht, and the Dutch HIV Monitoring Foundation.

***Financial support.*** This work was supported by the European Community Seventh Framework Programme (FP7-2007–2013) under grant number HEALTH-F3-2012–305578 and “AIDS Fonds” Netherlands (grant numbers 2008 026 and 2013 037).

***Potential conflicts of interest.*** All authors: No reported conflicts of interest. All authors have submitted the ICMJE Form for Disclosure of Potential Conflicts of Interest. Conflicts that the editors consider relevant to the content of the manuscript have been disclosed.
