# Supplementary material for: DC-SIGN Polymorphisms Associate with Risk of Hepatitis C Virus Infection Among Men who Have Sex with Men but not Among Injecting Drug Users
Source: J Infect Dis. 2017 Nov 13;217(3):353–7. doi: 10.1093/infdis/jix587 (PMC5853896; doi:10.1093/infdis/jix587)
Supplement: Supplementary Table S1 [file jix587_suppl_supplementary_table_s1.docx]

**Supplementary Table 1**

|  | MOSAIC | | ACS | |
| --- | --- | --- | --- | --- |
| **Characteristics** | **MEI** | **MEU** | **MEI** | **MEU** |
| **n (total=124)** | 32 | 30 | 22 | 40 |
| **Mean age ± SD** | 43.0 ±6.9 | 48.5 ±7.9 | 52.0 ±6.9 | 52.8 ±7.2 |
| **% Male gender** | 100% | 100% | 50% | 72.5% |
| **% Dutch Nationality** | 87.5% | 96.7% | 86.4% | 92.5% |
| **% HIV positive at entry** | 100% | 100% | 0% | 0% |
| **HIV seroconversion during follow-up** | n.a | n.a | 13.6% | 0% |
| **Median start date of Follow-up (IQR)** | 22/2/2011 (4/2/2010-2/8/2011) | 14/2/2011 (19/5/2010-20/12/2011) | 23/2/1988 (15/1/1987-08/02/1992) | 20/10/1992 (12/09/1988-22/04/1998) |
| **Median time of follow-up ± SD** | 4.01 ± 1.80 | 3.78 ± 1.30 | 14.96 ± 5.65 | 14.31 ±5.62 |
| **Mean duration IDU in years** | 4 IDU in last 6 months (no duration) | n.a | 7.21 ±3.42 | 8.45±4.83 |
| **% Reported sharing of needles^§^** | 0% | 0% | 75% | 55% |
| **Having an HCV-infected sex partner*** | 7 | 1 | n.a | n.a |
| **Fisting^§^** |  |  |  |  |
| **With steady partner** | 9 | 5 | n/a | n/a |
| **With casual partner(s)** | 10 | 8 | n/a | n/a |
| **Use of sex toys^§^** |  |  |  |  |
| **With steady partner** | 13 | 12 | n/a | n/a |
| **With casual partner(s)** | 15 | 4 | n/a | n/a |
| **Rectal bleeding during or after sex^§^** |  |  |  |  |
| **With steady partner** | ` | 10 | n/a | n/a |
| **With casual partner(s)** | 15 | 8 | n/a | n/a |
| **Groupsex^§^** | 24 | 23 | n/a | n/a |
| **Rectal bleeding during or after sex*** | 17 | 5 |  |  |
| **CD4 count last negative moment(cases)/last visit (controls)*** | 523±138 | 621±222 | n.a | n.a |
| **CD4 count nadir** | 277±160 | 269±179 | n.a | n.a |
| **Baseline Mosaic Risk score (medium)*^#^** | **2,9** | **1,1** |  |  |

n.a. = not applicable,

* = p < 0,05

**^§^** reported at least once

^#^ [10]
