# Supplementary material for: DC-SIGN Polymorphisms Associate with Risk of Hepatitis C Virus Infection Among Men who Have Sex with Men but not Among Injecting Drug Users
Source: J Infect Dis. 2017 Nov 13;217(3):353–7. doi: 10.1093/infdis/jix587 (PMC5853896; doi:10.1093/infdis/jix587)
Supplement: Supplementary Table S2 [file jix587_suppl_supplementary_table_s2.docx]

**Supplementary table 2^[[1]](#footnote-1)^**

| **Name** | **Orientation** | **Primer sequence 5'-> 3'** | **fragment length** |
| --- | --- | --- | --- |
| **L-SIGN repeat** | Fwd | CCTAAGTCAGGAACAATCCGA | 284bp, 353bp, 422bp, 491bp, 560bp, 629bp, 698bp (3/4/5/6/7/8/9 repeats, respectively) |
|  | Rev | GAACTCACCAAATGCAGTCTTCAAATC |  |
| **L-SIGN SNP rs2277998** | Fwd | GTCTAACTCCCAGCGGA | 45bp |
|  | Rev | TGGCAGGCGGTGACG |  |
| **DC-SIGN promotor PCR 1** | Fwd | GCAGTCTTGGTTCCTTGGAG | 630bp |
|  | Rev | ACTTGCAGTGCCTCCTCAGT |  |
| **DC-SIGN promotor PCR 2** | Fwd | TGCTGCTGTCCTCATTTTTG | 638bp |
|  | Rev | AGCATACAGAAACCCCGTTG |  |
| **Mutagenesis primer -139** | Fwd | TAGGGATCTGTCATCCAAAAGGCTAGTGGAAAGCATCAGAGCA |  |
|  | Rev | TGCTCTGATGCTTTCCACTAGCCTTTTGGATGACAGATCCCTA |  |
| **Mutagenesis primer -871** | Fwd | AGTACTAGTACATTTAATAACGTAGATAAATCTCACAAAACAG |  |
|  | Rev | CTGTTTTGTGAGATTTATCTACGTTATTAAATGTACTAGTACT |  |
| **Mutagenesis primer -939** | Fwd | CACACTGTAAGATTTGATTTTATGTGAATTTTGAGAACAGGCA |  |
|  | Rev | TGCCTGTTCTCAAAATTCACATAAAATCAAATCTTACAGTGTG |  |

1. **Amplification conditions:**

   L-SIGN repeat: denaturation at 95°C for 5 min, followed by 45 cycles at 95°C for 30s, 60°C for 30s and 72°C for 1 min and a final extension step at 72°C for 10 min.

   L-SIGN SNP rs2277998: 50°C for 2 min, denaturation at 95°C for 10 min, followed by 45 cycles at 95°C for 15s and 60°C for 15s, 72°C for 20s, followed by an HRM protocol of 95°C for 1 min, 40°C for 1 min and a fluorescence acquisition step at 60°C for 45s.

   DC-SIGN SNPs: denaturation at 95°C for 5 min, followed by 5 cycles at 94°C for 30s, 61°C for 30s (-0.5°C every cycle) and 72°C for 45s followed by 32 cycles at 94°C for 30s, 60°C for 30s and 72°C for 45s and a final extension step at 72°C for 10min. [↑](#footnote-ref-1)
