# Supplementary material for: DC-SIGN Polymorphisms Associate with Risk of Hepatitis C Virus Infection Among Men who Have Sex with Men but not Among Injecting Drug Users
Source: J Infect Dis. 2017 Nov 13;217(3):353–7. doi: 10.1093/infdis/jix587 (PMC5853896; doi:10.1093/infdis/jix587)
Supplement: Supplementary Table S3 [file jix587_suppl_supplementary_table_s3.docx]

**Supplementary Table 3**

| **MOSAIC** |  |  |  |  |  | **MEI vs MEU** |  |  |
| --- | --- | --- | --- | --- | --- | --- | --- | --- |
|  | genotype | MEI (n) | MEI (%) | MEU (n) | MEU (%) | OR | 95% CI | p value |
|  |  |  |  |  |  |  |  |  |
| **DC-SIGN -139** | AA | 3 | 11% | 2 | 25% |  | 0.105 - 1.30 | 0.09 |
|  | AG | 16 | 59% | 1 | 13% | 0.25 |  |  |
|  | GG | 8 | 30% | 5 | 63% | (GG vs AG+AA) |  |  |
| **DC-SIGN -871** | AA | 15 | 56% | 4 | 50% |  | 0.01 - 0.59 | < 0.01 |
|  | AG | 10 | 37% | 0 | 0% | 0.08 |  |  |
|  | GG | 2 | 7% | 4 | 50% | (GG vs AG+AA) |  |  |
| **DC-SIGN -939** | AA | 5 | 19% | 5 | 63% |  | 0.02 - 0.77 | 0.02 |
|  | AG | 16 | 59% | 0 | 0% | 0.14 |  |  |
|  | GG | 6 | 22% | 3 | 50% | (AA vs AG+GG) |  |  |
