# Supplementary material for: DC-SIGN Polymorphisms Associate with Risk of Hepatitis C Virus Infection Among Men who Have Sex with Men but not Among Injecting Drug Users
Source: J Infect Dis. 2017 Nov 13;217(3):353–7. doi: 10.1093/infdis/jix587 (PMC5853896; doi:10.1093/infdis/jix587)
Supplement: Supplementary Table S4 [file jix587_suppl_supplementary_table_s4.docx]

Supplementary Table 4

|  | **genotype n(%)** | | | | | | | | | | | |
| --- | --- | --- | --- | --- | --- | --- | --- | --- | --- | --- | --- | --- |
|  | 4/5 | 4/6 | 4/7 | 5 | 5/6 | 5/7 | 5/9 | 6 | 6/7 | 6/9 | 7 | 7/9 |
| **MEI** | 0 (0.0) | 0 (0.0) | 5 (9.6) | 2 (3.8) | 2 (3.8) | 9 (17.3) | 1 (1.9) | 1 (1.9) | 5 (9.6) | 1 (1.9) | 22 (42.3) | 4 (7.7) |
| **MEU** | 1 (1.5) | 1 (1.5) | 1 (1.5) | 6 (8.8) | 5 (7.4) | 13 (19.1) | 1 (1.5) | 3 (4.4) | 13 (19.1) | 0 (0.0) | 24 (35.3) | 0 (0.0) |

`
