# Supplementary material for: DC-SIGN Polymorphisms Associate with Risk of Hepatitis C Virus Infection Among Men who Have Sex with Men but not Among Injecting Drug Users
Source: J Infect Dis. 2017 Nov 13;217(3):353–7. doi: 10.1093/infdis/jix587 (PMC5853896; doi:10.1093/infdis/jix587)
Supplement: Supplementary Table S5 [file jix587_suppl_supplementary_table_s5.docx]

Supplementary Table 5

|  | homozygous | heterozygous | OR | p value |
| --- | --- | --- | --- | --- |
| **MEI** | 25 (48.1%) | 27 (51.9%) | 0.9820 | 0.9608 |
| **MEU** | 33 (48.5%) | 35 (51.5%) |  |  |
| **Total** | 58 (48.3%) | 62 (51.7%) |  |  |

`
