# Supplementary figures and images for: DC-SIGN Polymorphisms Associate with Risk of Hepatitis C Virus Infection Among Men who Have Sex with Men but not Among Injecting Drug Users
Source: J Infect Dis. 2017 Nov 13;217(3):353–7. doi: 10.1093/infdis/jix587 (PMC5853896; doi:10.1093/infdis/jix587)

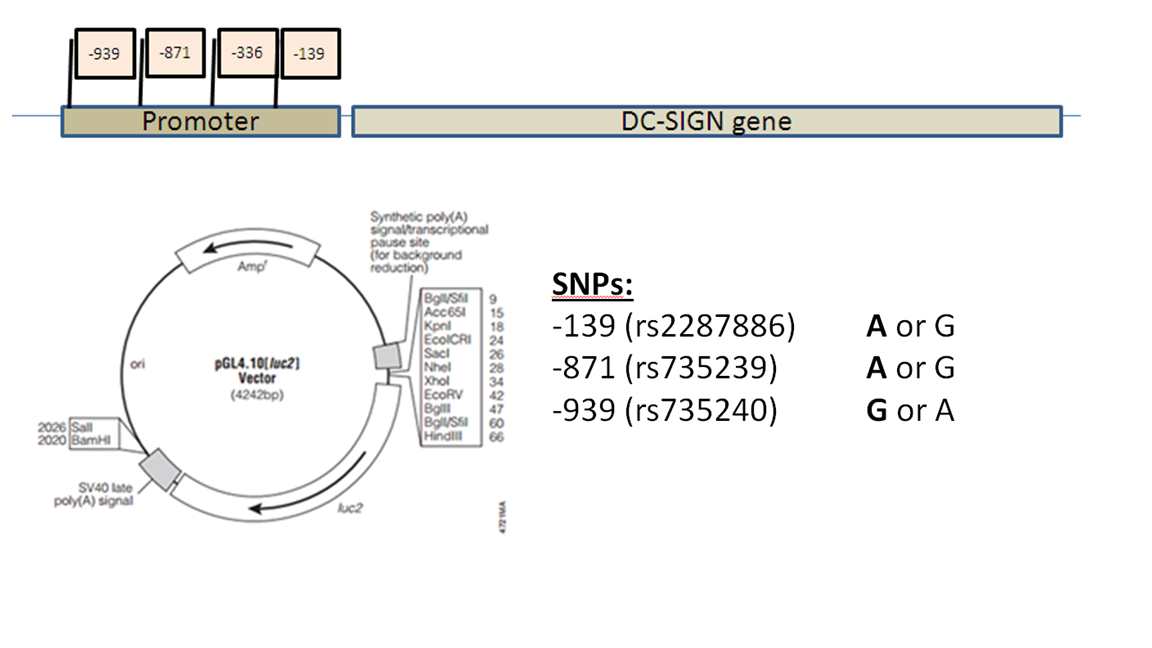

Supplement: Supplementary Figure S1 [file jix587_suppl_supplementary_figure_s1.png]
