# Supplementary material for: DC-SIGN Polymorphisms Associate with Risk of Hepatitis C Virus Infection Among Men who Have Sex with Men but not Among Injecting Drug Users
Source: J Infect Dis. 2017 Nov 13;217(3):353–7. doi: 10.1093/infdis/jix587 (PMC5853896; doi:10.1093/infdis/jix587)
Supplement: Supplemental Legends [file jix587_suppl_supplemental_legends.docx]

**Supplemental Table legends**

Table S1

Patients' characteristics from the MOSAIC and ACS cohorts. The MOSAIC behavioural risk factors is aggregated from multiple cohort visits.

Table S2

Primers and PCR conditions used for analysis of the DC/L-SIGN polymorphisms

**Table S3**

Distribution of DC-SIGN promoter SNPs based on genotype and MEU individuals with high MOSAIC risk scores (>=2.0) (>=2.0)

Table S4

Distribution L-SIGN repeat region among MEI and MEU individuals

Table S5

Zygosity L-SIGN repeat region compared between MEI and MEU individuals

No difference in L-SIGN zygosity between MEI and MEU individuals

**Supplemental Figure legends**

**Figure S1** Graphical representation of the DC-SIGN promoter and expression plasmid pGL4.10 construct
